# Triplex-quadruplex structural scaffold: a new binding structure of aptamer

**DOI:** 10.1038/s41598-017-15797-5

**Published:** 2017-11-13

**Authors:** Tao Bing, Wei Zheng, Xin Zhang, Luyao Shen, Xiangjun Liu, Fuyi Wang, Jie Cui, Zehui Cao, Dihua Shangguan

**Affiliations:** 1 Beijing National Laboratory for Molecular Sciences, Key Laboratory of Analytical Chemistry for Living Biosystems, CAS Research/Education Center for Excellence in Molecular Sciences, Institute of Chemistry, Chinese Academy of Sciences, Beijing, 100190 China; 2 University of Chinese Academy of Sciences, Beijing, 100049 China

## Abstract

Apart from the canonical Watson-Crick duplex, nucleic acids can often form other structures, e.g. G-quadruplex and triplex. These structures give nucleic acid additional functions besides coding for genetic information. Aptamers are one type of functional nucleic acids that bind to specific targets with high selectivity and affinity by folding into special tertiary structures. Despite the fact that numerous aptamers have been reported, only a few different types of aptamer structures are identified. Here we report a novel triplex-quadruplex hybrid scaffold formed by a codeine binding aptamer (CBA). CBA and its derivatives are G-rich DNA sequences. Codeine binding can induce the formation of a complex structure for this aptamer containing a G-quadruplex and a G·GC triplex, while codeine is located at the junction of the triplex and quadruplex. When split CBA into two moieties, codeine does not bind either moieties individually, but can bind them together by inducing the formation of the triplex-quadruplex scaffold. This structure formation induced by codeine binding is shown to inhibit polymerase reaction, which shows a potential application of the aptamer sequence in gene regulations.

## Introduction

**Figure 1:**
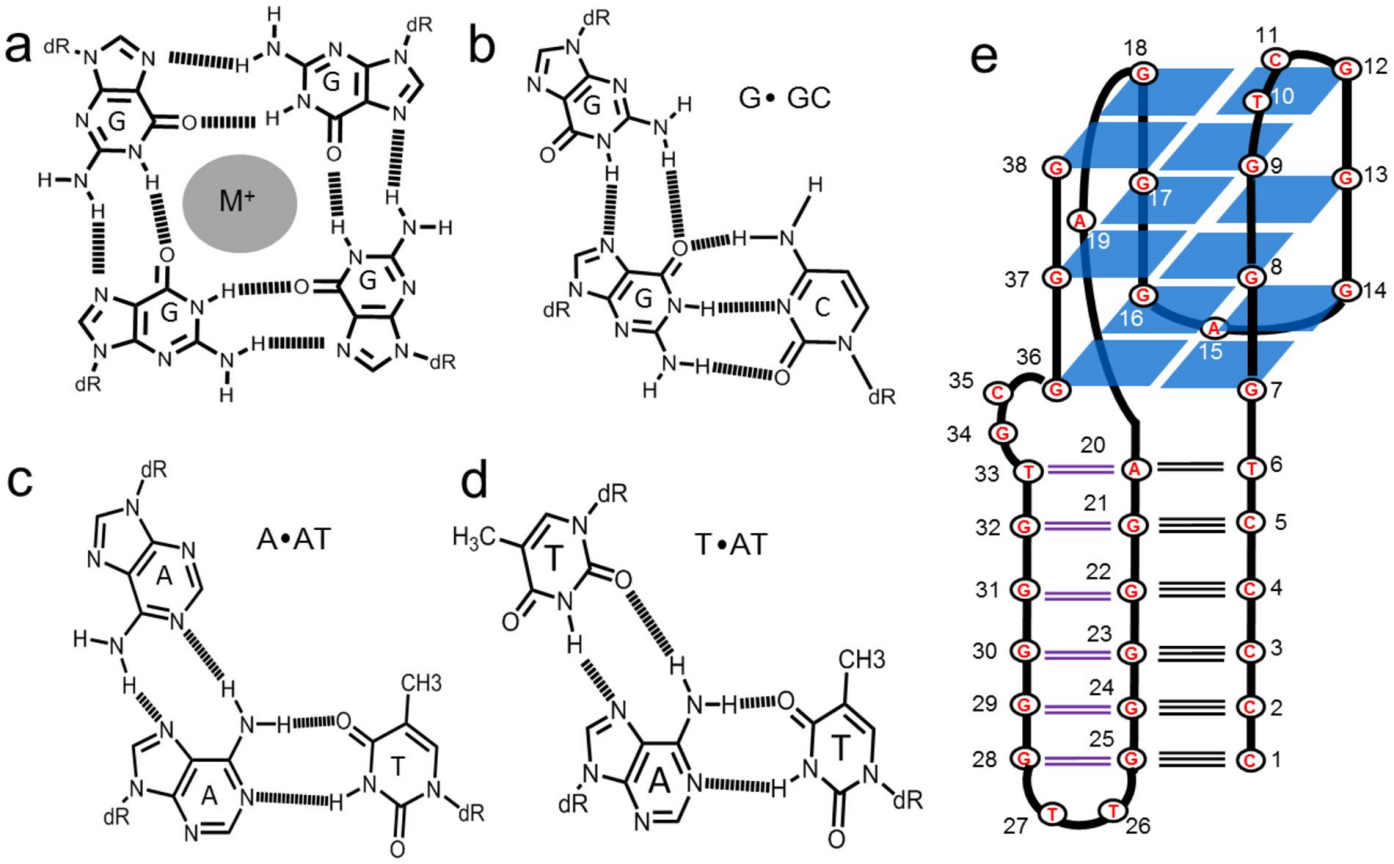
(**a**) Structure of G-quartet; (**b**) Structure of G·GC triad; (**c**) Structure of A·AT triad; (**d**) Structure of T·AT triad; (**e**) The schematic depiction of predicted scaffold 1 of CBA-1.

Nucleic acid sequences can form many non-canonical structures other than the Watson-Crick duplex structure (e.g. pseudoknot, quadruplex, triplex and i-motif) through base-pairing interactions, such as Watson-Crick base pairing, wobble base pairing and Hoogsteen base pairing. These structures are found in various functional nucleic acids, such as transfer RNA, riboswitches, DNAzymes, ribozymes, aptamers, and many nanodevices^1–5^. Among these structures, G-quadruplexes are four-stranded structures formed by repetitive G-rich nucleic acid sequences. They have attracted considerable interest because of their important biological functions and applications in biosensors and nanotechnology^6^. G-quadruplexes are composed of planar arrangements of four guanine-bases stabilized by eight Hoogsteen hydrogen bonds (known as G-quartet) (Fig. 1a)^7^. This structures are widely considered to play important roles in regulation of many cellular functions, such as telomere maintenance^8^, transcription^9,10^ and translation^11,12^. Triplexes are triple helical structures, in which the third strand binds to a homopurine strand of the Watson-Crick duplex within the major groove^13^. There are two different triplex motifs, purine motif (R) and pyrimidine motif (Y). In the R motif, the third strand binds to the duplex purine strand in an antiparallel orientation via reverse-Hoogsteen hydrogen bonds and forms A**·**AT, T**·**AT, and G**·**GC base-triplets (Fig. 1b–d)^14,15^. In the Y motif the third strand binds to the duplex purine strand in a parallel orientation via Hoogsteen hydrogen bonds and forms T·AT and C·GC base-triplets^14^. Potential triplex-forming oligonucleotides are found throughout genomic DNA^14,15^ and RNA^16,17^, and are also considered to play important roles in gene transcription and expression^18^.

Aptamers are single strand oligonucleotides (DNA or RNA), generated *in vitro* using the SELEX technique. They can bind to various targets with high affinity and specificity, with potential applications in affinity separation, biosensors, drug discovery, and diagnosis^1,19–21^. The specific binding of aptamers results from their well-defined tertiary structures composed of simple secondary structures, such as hairpins, bulges, pseudoknots, T-junctions, G-quadruplexes, and their combination^22,23^. These complex tertiary structures create well-defined binding shapes, facilitating the formation of electrostatic interactions, hydrogen bonds and π-π stacking interactions between aptamers and their targets. The structural elucidation of aptamers can provide insights into the principles, patterns and diversity associated with nucleic acid architecture, molecular recognition and the adaptive binding^24^. Apart from stems, loops, and bulges, G-quadruplexes are often found in aptamer, and act as the binding motif of many aptamers^25–30^. In contrast, triplexes have been rarely reported in aptamers.

Herein we report a novel aptamer structure composed of a G-quadruplex and a triplex. The binding features of this codeine binding aptamer (CBA) to codeine^31^ were investigated. We also show that the formation of the triplex-quadruplex scaffold induced by codeine can lead to inhibition of DNA polymerase activity.

## Results and Discussion

### The potential structure of codeine binding aptamer

The original CBA (CBA-0) is a highly G-rich sequence generated in our lab previously, which contains six guanine (G) tracts and a cytosine (C) tract (5C-T-3G-TC-3G-A-3G-AA-5G- TT-5G-TGC-2G) (Table S1). G-rich sequences often fold into G-quadruplexes. The formation of a G-quadruplex motif only needs four G-tracts, the extra G-tracts in a G-quadruplex-forming sequence act as loops^32,33^. Usually, a sequence containing more than four G-tracts may form multiple G-quadruplexes in solution. In the case of CBA-0, there are a 5C-tract and two 5G-tracts, which have the potential to fold into a perfect G·GC triplex structure. In addition, previously, we have found that removal of the 2G-tract at 3′-end of CBA-0 causes the loss of its binding ability to codeine^31^, suggesting that the 2G-tract may be involved in the formation of a G-quadruplex together with the other three 3G-tracts. As a result, CBA-0 may adopt a combination of triplex and G-quadruplex. Based on this hypothesis, the G-quadruplex moiety of CBA-0 likely forms an imperfect G-quadruplex structure because of the three 3G-tracts and one 2G-tract. A new sequence, CBA-1, was therefore designed by adding a G-base on the 3′-end of CBA-0 so that it can form a perfect G-quadruplex with four 3G-tracts. Because our previously study found that CBA-0 tightly bound codeine in buffers with high concentration of Na^+^ (> 50 mM) and low concentration of K^+^ (≤ 50 mM)^31^, unless otherwise indicated, all the experiment in this paper were performed in phosphate buffer saline (PBS, 140 mM NaCl + 2.5 mM KCl + 1.6 mM KH_2_PO_4_ + 15 mM Na_2_HPO_4_, pH 7.4) with 2.5 mM MgCl_2_. CBA-1 was measured to have a much higher binding affinity (K_d_ = 0.07 ± 0.01 μM) to codeine than that of CBA-0 (K_d_ = 0.25 ± 0.08 μM) by isothermal titration calorimetry (ITC) (Figure S1), which suggests that a stable G-quadruplex moiety is critical for codeine binding.

Because the orientation of the three strands in G**·**GC type triplex is fixed, the G_21-25_ tract of CBA-1 inclines to form a Watson-Crick duplex with the 5 C tract, and the G_28-32_ tract acts as the third strand in an antiparallel orientation so that G_36-38_ and G_7-9_ can locate on the same side for the formation of G-quadruplex. G-quadruplexes can be classified into parallel, anti-parallel and hybrid structures based on the arrangement and orientation of the strands, therefore, CBA-1 may adopt five possible structures with hybrid G-quadruplex (scaffold 1 and 2), parallel G-quadruplex (scaffold 3) or antiparallel G-quadruplex (scaffold 4 and 5) (Figs 1e and S2).

### Circular dichroism spectra and binding affinity

**Figure 2:**
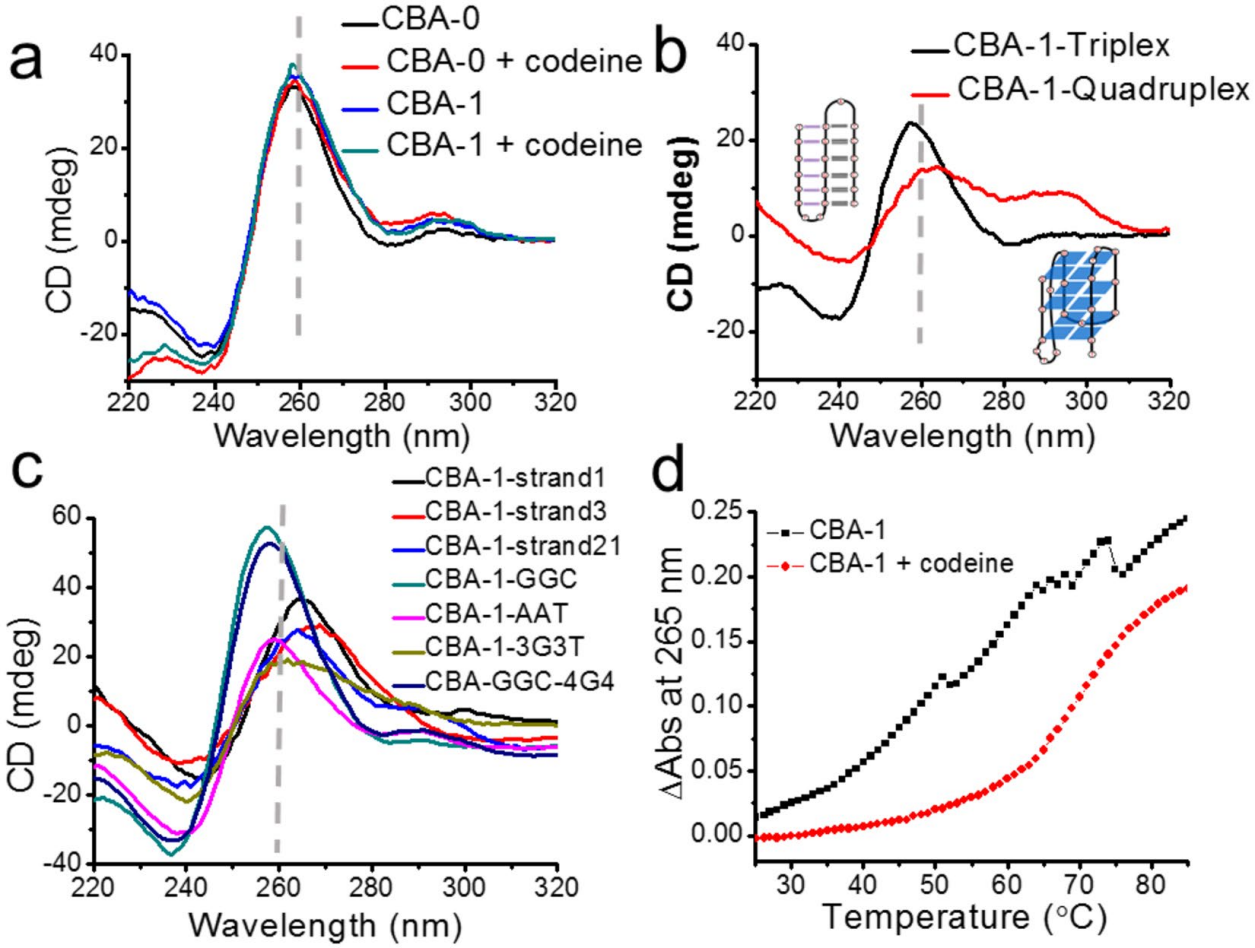
(**a**) CD spectra of CBA-0 and CBA-1 (4 µM) in the absence or presence of codeine (4 µM). (**b**) CD spectra of CBA-1-triplex and CBA-1-quadruplex (4 µM). (**c**) CD spectra of derivative sequences of CBA-1 (4 µM). (**d**) The melting curve of CBA-1 (4 µM) in the absence or presence of codeine (8 µM) (absorbance at 265 nm).

Circular dichroism (CD) spectra are commonly used to provide a signature profile of the secondary structure of a given DNA. The intramolecular triplex DNA displays a negative peak around 240 nm, a positive peak around 257 nm and a second negative peak around 280 nm in CD spectra^15^. For G-quadruplexes, the antiparallel structure has a positive CD peak around 295 nm and a negative peak around 260 nm; the parallel structure has a positive peak around 264 nm and a negative peak around 240 nm; and hybrid structures show positive signals around 295 and 265 nm and a negative peak around 240 nm^7^. The CD spectra of CBA-0 and CBA-1 (Fig. 2a) showed a positive main peak near 260 nm, and a small positive peak near 290 nm (combined signal of triplex and G-quadruplex); the addition of codeine caused minor change of their CD spectra. Due to the spectral overlap of triplex and parallel/hybrid G-quadruplexes in CD spectra, structural information of triplex and quadruplex was difficult to be elucidated from the CD spectra of the CBAs. To solve this problem, CBA-1 was split into two moieties, G-quadruplex and triplex (Table S1), and their CD spectra were collected. As shown in Fig. 2b, the CBA-1-triplex showed the typical CD signals of a triplex. And CBA-1-quadruplex showed the CD signals of a hybrid structure (3 + 1) of G-quadruplex that contained three parallel and one antiparallel strands (a positive main peak at 265 nm, a shoulder peak near 290 nm and a negative at 240 nm)^34^.

In order to demonstrate whether the triplex and G-quadruplex structures were essential for the binding of CBA, mutation assay was performed. The triplex moiety of CBA-1 was mutated by replacing the 5C-tract with an 5A-tract (CBA-1-strand1), replacing a 5G-tract (G_28-32_, the third strand of the triplex) with TATAT (CBA-1-strand3), or interchanging the 5C-tract and a 5G-tract (G_21-25_) (CBA-1-strand21). The CD spectra of these sequences showed that their main CD peaks shifted from 257 nm to around 265 nm, while the negative CD peak at 280 nm disappeared, suggesting the loss of triplex structure (Fig. 2c). These mutations also caused the sequences to lose binding ability to codeine (Figure S3). As expected, extending the triplex of CBA-1 with three G**·**GC triads (CBA-1-GGC) or three A·AT triads (CBA-1-AAT) did not cause any CD signal shift (Fig. 2c), and kept the binding to codeine (K_d_ = 0.05 ± 0.02 and 0.07 ± 0.03 μM, respectively) (Figure S4). These results suggest that the triplex moiety is essential for the target binding of CBA.

When replacing all the 3G-tracts of CBA-1 with 3T-tracts, the resulted sequence CBA-1-3G3T lost binding ability to codeine (Figure S3d), and showed a broad positive CD signal with the maximum at 257 nm (combined signal of triplex and single strand) (Fig. 2c). In addition, extending the G-quadruplex moiety of CBA-1-GGC to form a four-layer quadruplex (CBA-1-GGC-4G4) only caused a very small shift of the main positive CD signal but led to reduced binding ability (K_d_ = 0.30 ± 0.17 μM) (Figs 2c and S4c), suggesting that the added quartet did not destroy the conformation of the aptamer but might have slightly affected the binding pocket. These additional results confirmed that G-quadruplex plays an important role for codeine binding. Since no significant peak at 295 nm was found in CD spectra of all the CBA sequences, their G-quadruplex moiety is not likely to adopt an antiparallel structure, i.e. scaffold 4 and 5 may not be the structure of CBA-1.

The influence of codeine on the structural stability of CBA-1 was investigated by thermal denaturation. The melting curve of CBA-1 showed several transitions in the absence of codeine, but only one transition in the presence of codeine and with a significant increase of melting temperature (Fig. 2d). This finding suggests that codeine induced the formation of a unique and more stable structure.

### G-quadruplex probe competition assay

In order to further confirm the G-quadruplex in CBA-1, a fluorescent G-quadruplex probe, BPBC, that binds to G-quadruplexes through end-stacking on G-quartet surface was used to compete with codeine^35^. BPBC emit a very weak fluorescence in the absence of G-quadruplex. After mixing with CBA-1 or CBA-1-strand1 (non-binding sequence without the predicted triplex moiety), the fluorescence of BPBC increased approximately 200 fold (DNA:BPBC = 1:1) and 400 fold (DNA:BPBC = 1:3) (Figure S5), indicating the presence of G-quadruplex in both sequences. The addition of equivalent amount of codeine and BPBC to CBA-1 (DNA:BPBC:codeine = 1:3:3) resulted in a fluorescence intensity about half of that from the mixture of DNA/BPBC at 1:3 ratio. Interestingly, further addition of codeine (DNA:BPBC:codeine = 1:3:10) did not continue to cut down BPBC fluorescence. One possible explanation is that codeine only bound at one end of G-quadruplex moiety of CBA-1 while BPBC could bind at both ends through end-stacking. Compared to CBA-1, the addition of codeine did not affect the fluorescence of CBA-1-strand1/BPBC solution (DNA:BPBC:codeine = 1:3:3), indicating codeine binding did not happen without the triplex structure. These results suggest that codeine most likely binds to only one end of the G-quadruplex that is adjacent to the triplex structure, which agrees with the ITC results that CBA only binds one codeine.

### Structure study by 1H-NMR

**Figure 3:**
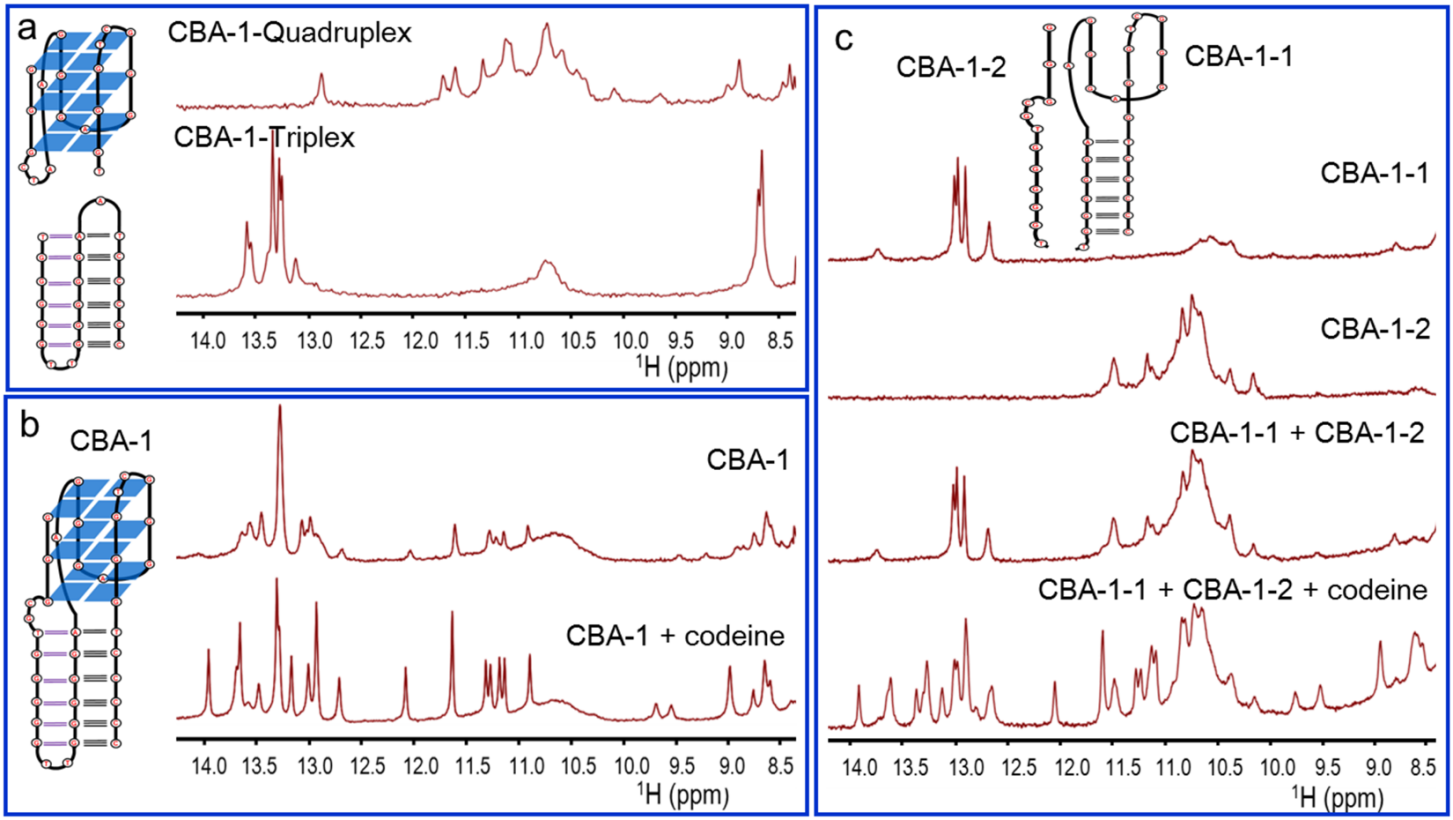
DNA sequences related to CBA-1 and their 1D ^1^H NMR spectra in the range of 8.4–14.5 ppm.

In order to further confirm the triplex-quadruplex scaffold of CBA, ^1^H-NMR spectra were collected. Guanines in the tetrads of G-quadruplexes exhibit characteristic chemical shifts around 10–12 ppm, which are assigned to the imino protons in Hoogsteen hydrogen bonding^36–38^. In triplex, the hydrogen bonded imino protons of thymine and guanine exhibit characteristic chemical shifts in the range of 12.5–14.5 ppm in addition to those expected from the Watson-Crick duplex, and the cytosine amino protons display a set of downfield shifted resonances around 9 ppm^15,38,39^. The ^1^H-NMR spectra of the split triplex and quadruplex moieties of CBA-1 (CBA-1-Quadruplex and CBA-1-Triplex) showed a group of signals in the range of 10–12 ppm and 13–14 ppm respectively (Fig. 3a), which correspond to the imino proton signals of quadruplex and triplex. CBA-1-Triplex also showed strong ^1^H-NMR signals around 8.7 ppm corresponding to cytosine amino protons in triplex. In the absence of codeine, CBA-1 displayed similar ^1^H-NMR signals with low resolution in the range of 13–14 ppm and 8.6–8.8 (Fig. 3b), suggesting the presence of triplex. It also showed broad envelopes and some small peaks in the range of 10–12 ppm, which suggests that CBA-1 might form multiple quadruplex species. In contrast, in the presence of codeine, CBA-1 showed well-resolved NMR signals of triplex and quadruplex, suggesting that codeine caused CBA-1 to form a well-defined and more stable unique triplex-quadruplex hybrid scaffold. Similar results were also obtained from other codeine binding sequences (CBA-0, CBA-1-GGC and CBA-1-AAT) (Figures S6 and S7). The non-binding sequences (CBA-1-strand1, CBA-1-strand3, CBA-1-strand21 and CBA-1-3G3T) did not show well-resolved NMR signals of triplex or quadruplex whether in the presence or absence of codeine (Figure S8).These results further confirm that a well-defined unique triplex-quadruplex scaffold is essential for the aptamer to bind codeine. In addition, compared to CBA-1-Quadruplex, the ^1^H-NMR peaks of all codeine-binding sequences in the range of 10–10.8 disappeared, and only 8 imino well-dissolved peaks of G-quadruplex were observed in the presence of codeine, suggesting that the quadruplex moiety of these sequences only contains two layers of G-tetrads, i.e. the binding of codeine might preclude the formation of G-quadruplex with three layers of G-tetrads.

Since high concentration of K^+^ was found unfavourable to the binding of CBA and codeine^31^, we further invesigated the effect of 500 mM K^+^ on the structure of CBA-1 by ^1^H-NMR spectra. In the buffer only containing 500 mM K^+^ (without Na^+^), CBA-1 showed broad NMR signals of G-quadruplex (10–12 ppm), but did not show any signals of triplex in the range of 13–14 ppm whether in the absence or presence of codeine (Figure S9). In the buffer without K^+^ (only Na^+^), the ^1^H-NMR spectra of CBA-1 were very similar with those in PBS buffer (containing 4.1 mM K^+^) in the absence or presence of codeine. After adding 500 mM KCl in the buffer containing Na^+^ and codeine, the well-resolved signals of triplex (13–14 ppm) disapeared and the signals of G-quadruplex became broad (Figure S10). These results suggest that high concentration of K^+^ leaded CBA-1 to form multiple G-quadruplexes and disturbed the formation of G**·**GC triplex structure.

G-rich sequences can simultaneously fold into many quadruplex species in solution, and the populations of the various structures are present in a dynamic equilibrium under any given conditions^40^. In the absence of codeine, CBA sequences formed multiple intermolecular and intramolecular structures, which could have resulted in the low-resolution NMR signals and the multiple transitions of the melting curve (Fig. 2d). The binding of codeine stabilized one of the structures (binding structure), and moved the equilibrium toward the formation of a unique binding structure, resulting in the well-defined NMR signals, as well as the single transition of the melting curve and a much higher T_m_ value.

Based on the predicted structures (Figs 1e and S2), the 12-nt long sequence at the 3′-end of CBA-1 caught our attention. This part contains the third strand of triplex and the forth strand of G-quadruplex. The melting of triplexes has shown that the third strand dissociates at a much lower temperature than that of the dissociation of the core duplex^41–43^. The G-triplex structure, a stable folding intermediate of the G-quadruplex formed by G:G:G triad planes has also been proved^44^. Therefore, it is possible that the 3′ end of CBA sequences may open and close in the absence of codeine. The assumption was further proved by DNA-sequence splitting. CBA-1 was split into a 12-nt sequence (CBA-1-2) and a 26-nt sequence (CBA-1-1), and their binding of codeine was measured with codeine-modified beads (Figure S11). The split sequences did not bind codeine separately, but bound it together. Similar result was also obtained in split CBA-1-GGC. The melting assay showed that codeine greatly increased the T_m_ value (∆T_m_ ≈ 4 °C) of the mixture of CBA-1-1/CBA-1-2, indicating the formation of a stable complex (Figure S12). In addition, the ^1^H-NMR spectrum of CBA-1-1 showed strong signals around 13 ppm (Fig. 3c), which were expected from the Watson-Crick duplex. And CBA-1-2 showed a group of ^1^H-NMR signals in the range of 10–12 ppm, which might be due to the formation of intermolecular quadruplexes. In the absence of codeine, the ^1^H-NMR spectrum of the CBA-1-1/CBA-1-2 mixture was simply the assembly of both individual spectra, implying no new structure was formed. Interestingly, after addition of codeine, well-resolved NMR signals similar to those of CBA-1/codeine complex appeared in the ^1^H-NMR spectrum of CBA-1-1/CBA-1-2 mixture (Fig. 3c), suggesting that codeine brought both sequences together to form the triplex-quadruplex scaffold as with CBA-1.

### Dimethyl sulfate (DMS) footprinting assay

**Figure 4:**
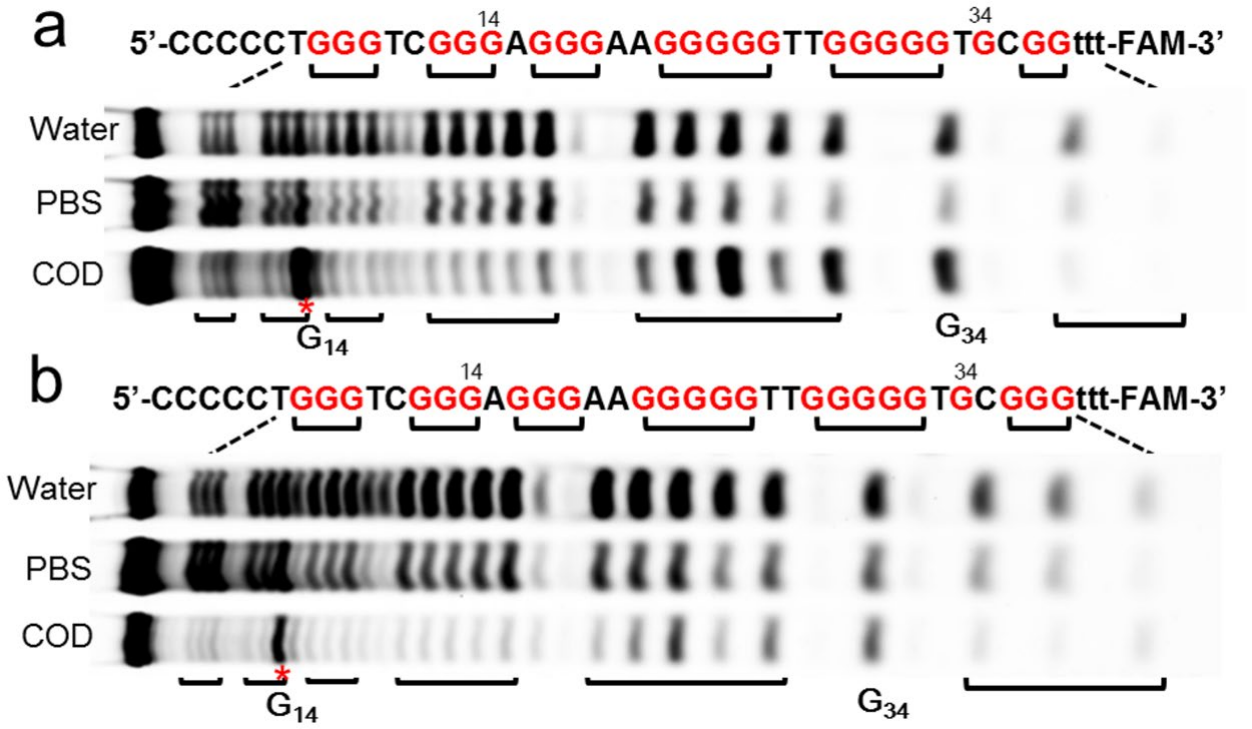
Cleavage fragments of CBA-0 (**a**) and CBA-1 (**b**) resolved by denaturing gel electrophoresis; Lane water: DNA cleaved in water; Lane PBS: DNA cleaved in binding buffer; Lane COD: DNA cleaved in binding buffer containing codeine. The full-length gel is presented in Supplementary Figure S19.

DMS footprinting assay can probe the accessibility of N7 of guanine in G-quadruplex and triplex structure^45,46^. N7 of guanine in G-quartet and N7 of the second guanine of G**·**GC triplet are involved in hydrogen bonding, which were generally evenly protected from methylation. In order to further illustrate the structure of CBA sequences, DMS experiments were performed. As shown in Fig. 4, CBA sequences did not form stable triplex-quadruplex structure in water, and almost all G bases in CBA-0 and CBA-1 were not protected as a result. Compared with in water, the G_12–13_, G_16–18_, G_21–25_, G_28–32_, G_34_ and G_36_ of CBA-0 were partly protected from methylation in PBS buffer, suggesting these G-bases were involved in the formation of triplex or G-quadruplex. Upon binding with codeine, G_7–9_, G_12–13_, G_16–18_, G_21–25_ and G_35_ of CBA-0 were further protected, but G_28–32_ and G_34_ became less protected, which agreed well with the formation of the triplex-quadruplex structure. Similar result was obtained in the DMS experiment of CBA-1 (Fig. 4b). In the absence of codeine (PBS buffer), the G-bases in quadruplex and in the second G-strand (G_21–25_) of triplex of CBA-0 and CBA-1 were less protected than those in the presence of codeine, which may be due to the structural diversity of both sequences in the absence of codeine, e.g. the opening and closing of the 3′ end part as discussed earlier. Interestingly, G_14_ in both sequences was not protected and even more hypersensitive to DMS methylation when binding to codeine, indicating that this G-base was exposed to DMS methylation and not involved in G-quadruplex formation. This is also in agreement with the NMR result that the G-quadruplex moiety contains two layers of G-tetrads after binding codeine.

### The binding site study

The binding site in CBA was investigated by mutation assay. As G_14_ and G_34_ of CBA-1 were not protected from methylation in DMS assay, G_14_ of CBA-1 was replaced by A (CBA-1-G14A). The mutated sequence still showed high binding ability to codeine (Figure S13). The ^1^H-NMR spectra of CBA-1-G14A in the presence of codeine also showed well-resolved and sharp signals of triplex and quadruplex (Figure S14). These results further confirmed that G_14_ of CBA-1 was not involved in the formation of G-quadruplex after codeine binding, and might not interact with codeine directly. Mutating G_34_ of CBA-1 with T (CBA-1-G34T) greatly decreased its binding ability. Removing G_34_ (CBA-1-G34R) or G_34_C_35_ (CBA-1-G34C35R) caused the loss of binding ability totally, but did not affect the CD spectra of these sequences (Figures S13, S15), suggesting that G_34_C_35_ played a key role in codeine binding. Since G_34_C_35_ is located at the junction between G-quadruplex and triplex, codeine is likely bound in the pocket formed by G_34_C_35_, G-quadruplex and triplex.

2-aminopurine (2-AP), an adenine analog with high intrinsic fluorescence, usually serves as a versatile, site-specific conformational reporter of nucleic acids^47^. The fluorescence of 2-AP is sensitive to its environment, e.g. quenched by stacking with other bases or molecule^48^. In order to further study the conformation, G_14_, A_15_, A_19_ and A_20_ of CBA-1 were replaced with 2-AP respectively (CBA-1-A14AP, CBA-1-A15AP, CBA-1-A19AP and CBA-1-A20AP). The mutated sequences remained their binding ability to codeine except for CBA-1-A19AP whose binding ability was greatly decreased (Figure S16). The fluorescence intensity of CBA-1-A14AP and CBA-1-A15AP in the absence of codeine was much higher than that of CBA-1-A19AP and CBA-1-A20AP (Figure S17), suggesting that AP_14_ and AP_15_ were free of interaction with other bases. Codeine binding to CBA-1-A14AP, CBA-1-A15AP and CBA-1-A20AP partly quenched their fluorescence, suggesting that codeine binding might have influenced the local environment of AP_14_, AP_15_ and AP_20_, i.e. these bases are near the binding pocket. According to these results, the predicted scaffold 2-3 may not the binding structure of CBA. Since replacing A_19_ of CBA-1 with 2-AP (CBA-1-A19AP) greatly decreased its binding ability, this A_19_ was further replaced with T or G (CBA-A1-19T and CBA-1-A19G), which also caused great drop of the binding ability to codeine for both sequences (Figure S16). The CD spectra of CBA-1-A19AP, CBA-1-A19G and CBA-1-A19T showed that their main CD peaks shifted from 261 nm to 257 nm (Figure S18), suggesting that A_19_ is essential for maintaining the quadruplex structure. All the above results suggest that the predicted scaffold 1 is the most likely binding structure of CBA-1.

### Molecular modeling and docking

**Figure 5:**
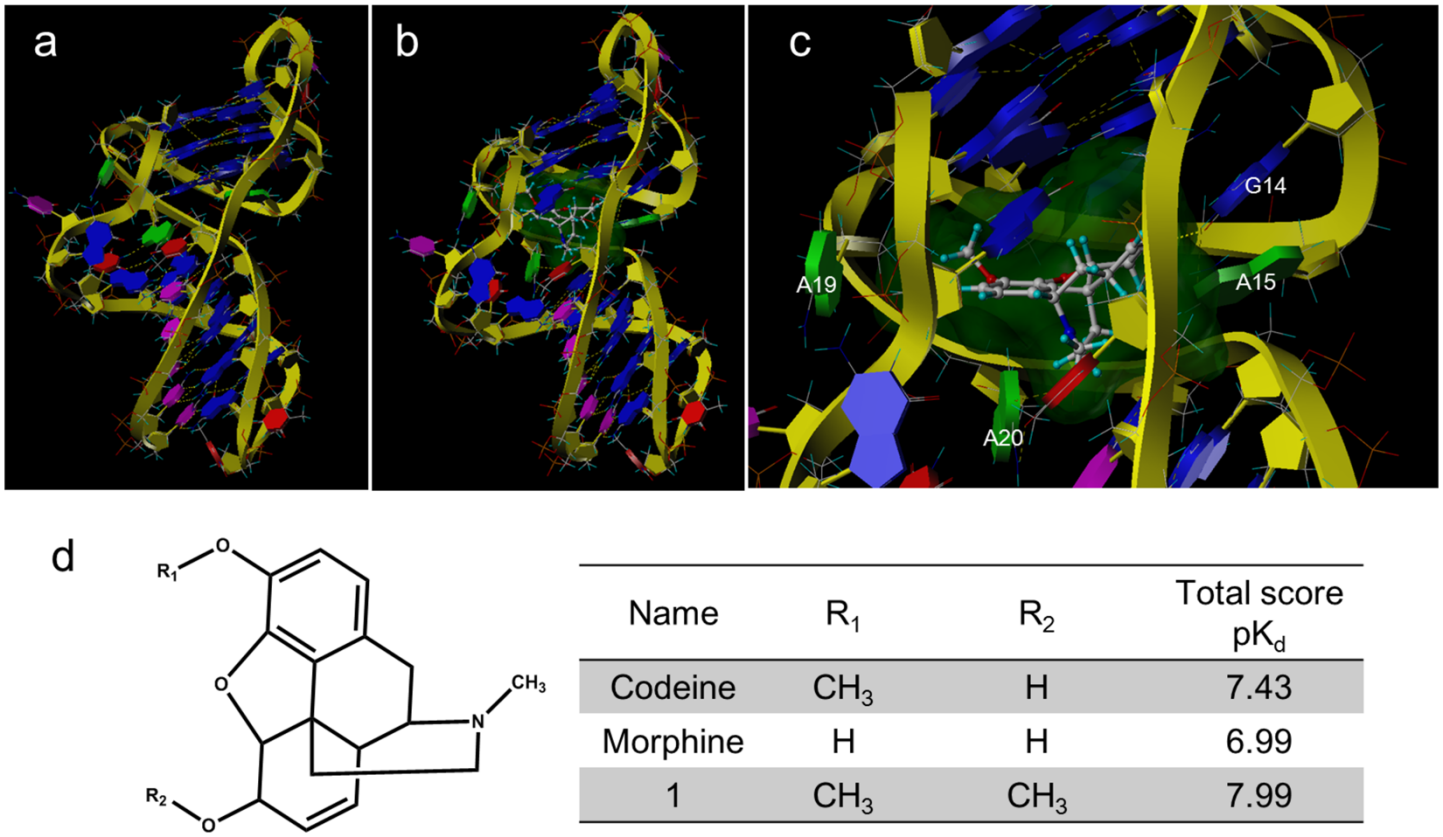
(**a**) The predicted molecular model of CBA-1. (**b**) The predicted binding model of codeine to CBA-1 by molecular docking. (**c**) The detailed binding pocket of CBA-1 with codeine. The predicted binding pocket was shown as green. (**d**) The docking total score of codeine analogues.

Because we failed to obtain the single crystal of CBA-1/codeine complex for X-ray diffraction analysis; and failed to obtain the detail structure of CBA-1/codeine complex through 2D NMR^34^; we tried to understand the binding between codeine and CBA-1 through molecular docking using Sybyl X 2.0. Based on all of the results above, the binding structure of CBA-1 is most likely a triplex-quadruplex hybrid structure, in which the G-quadruplex moiety of the predicted scaffold 1 is similar to the (3 + 1) G-quadruplex form 2 of human telomere sequence^37,49^. The starting structure of CBA-1 was therefore generated by combining the reported NMR structures of (3 + 1) G-quadruplex (PDB entries 2JPZ)^50^ and the G·GC triplex structures (PDB entries 134D)^39^. This molecular model was then energy-minimized using the Tripos force field with Gasteiger-Marsili charges (Fig. 5a–c). The binding site is located in the junction between the triplex and G-quadruplex, and G_14_, A_15_, A_19_ and A_20_ are near the binding pocket. Molecular docking was performed using Surflex-Dock program, with the docking scores expressed in –lgK_d_ units, where K_d_ is the dissociation constant. The docking result showed that codeine bound to CBA-1 with a score of 7.34. According to the equation ΔG = −RTlnK, ΔG was calculated to be −41.87 kJ/mol, which was very close to that measured by ITC (−40.9 ± 0.5 kJ/mol), suggesting that the binding was very stable. In the presence of codeine, the G-quartet plane facing the binding pocket was destroyed by the binding of codeine, which agreed with the NMR and DMS results that the G-quadruplex moiety contains two layers of G-tetrads. Furthermore, two codeine analogues, morphine and methyl substituted codeine (compound 1) were also docked into the binding pocket (Fig. 5d). Morphine showed a slightly weaker binding affinity than codeine (score: 6.99 vs 7.43), which is consistent with our previous results^31^. As a comparison, Methyl substituted codeine displayed higher affinity (score: 7.99) to CBA-1 than codeine, suggesting the substitution at the hydroxyl group of codeine did not affect the aptamer binding. This result is expected since CBA was selected using codeine immobilized beads by SELEX technique, in which codeine was linked to beads through this hydroxyl group.

### Polymerase Stop Assays

**Figure 6:**
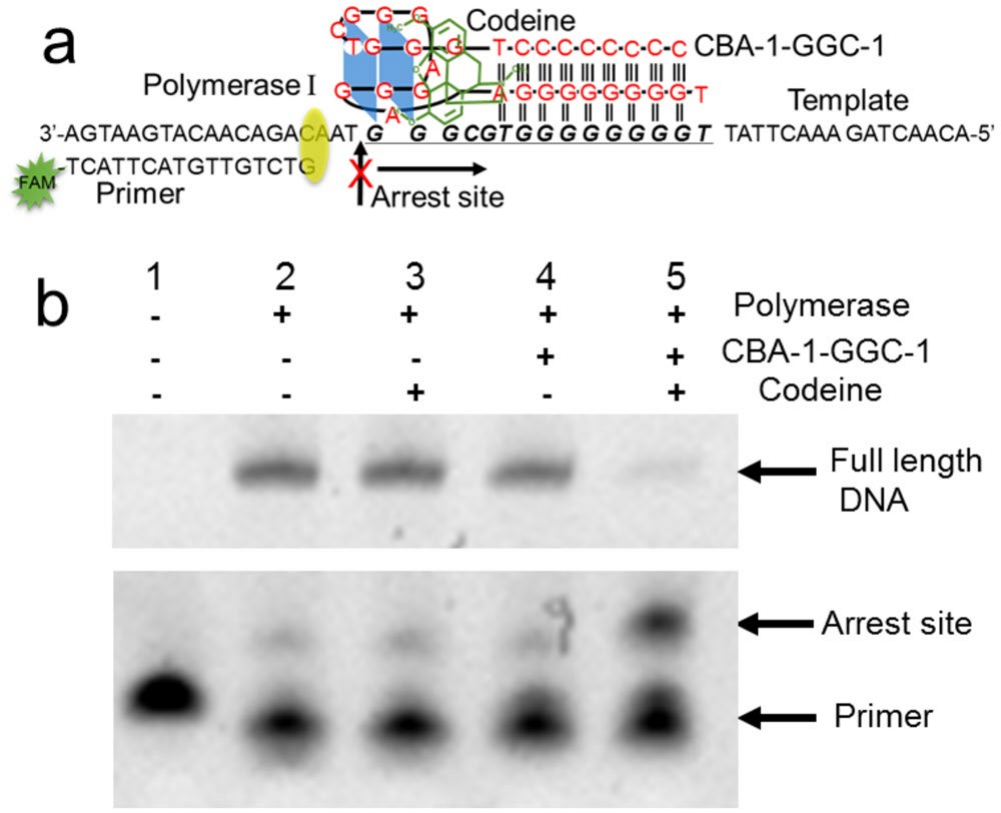
(**a**) The principle of the polymerization arrest caused by triplex-quadruplex hybrid scaffold formation. (**b**) The gel electrophoresis of the extension product by polymerase I. DNA polymerization was arrest at the triplex-quadruplex scaffold induced by codeine. The full-length gel is presented in Supplementary Figure S20.

Both triplex and G-quadruplex are reported to form in cells and repress gene transcription^51,52^. They are widely utilized as tools to regulate gene expression and study the molecular mechanisms of DNA repair, recombination, and mutagenesis^53,54^. However, a few factors may limit their application. For example, these structures form spontaneously in the presence of a physiological amount of monovalent cations^55,56^; G-rich sequences often form multiple inter/intra-molecular structures; and the triplex structures are much less stable than duplex and G-quadruplex^57^. In comparison, the CBA/codeine system may have some notable advantages because of the unique and stable structure induced by codeine. Since we have shown that codeine could induce the formation of triplex-quadruplex scaffold of the split CBA sequences, we have tested the feasibility of using codeine to control gene express by performing a DNA polymerase I stop assay. The DNA template contained the 3′end sequence of CBA-1-GGC (CBA-1-GGC-2, T-8G-TGC-3G); DNA polymerase I (Klenow Fragment) was used to extend primer sequence; sequence CBA-1-GGC-1 (the other part of CBA-1-GGC) was used to form the triplex-quadruplex structure with CBA-1-GGC-2 in the template (Fig. 6a). As shown in Fig. 6b, codeine or CBA-1-GGC-1 alone did not affect the extension of primer, but codeine and CBA-1-GGC-1 together almost totally halted the extension, indicating that codeine likely induced the formation of the triplex-quadruplex structure that blocked polymerase progression. Unlike many organic ligands that bind to most G-quadruplexes, codeine is highly specific for CBA^31^. These results may indicate great potentials for precise regulation of gene functions by using CBA sequences and codeine.

In summary, the binding structure of CBA has been investigated and a novel triplex-quadruplex scaffold has been proposed and confirmed. CBA and its derivatives are G-rich DNA sequences containing six G-tracts and a C-tract, which prefer to form structures with G-quadruplex and G·GC based triplex. The binding of codeine induces CBA to form a well-defined triplex-quadruplex scaffold, and greatly enhances their thermal stability. The codeine binding pocket is located in the cavity of the junction formed by the triplex and quadruplex. When split in two, the two individual parts of CBA cannot bind codeine on their own, and can only bind codeine when both are present. A polymerase stop experiment showed that the split sequences could form the triplex-quadruplex scaffold induced by codeine, resulting in blocking of the polymerase reaction. This novel scaffold not only has implication in rational design of aptameric sensors, but also has the potential to function as regulators of gene expression. Although the detailed structure of CBA cannot been elucidated currently, the triplex-quadruplex hybrid scaffold offers new insights in DNA architecture and molecular recognition.

## Materials and Methods

### Materials

Synthetic oligodeoxynucleotides were obtained from Sangon Biotech Co. Ltd. (Shanghai, China) or Zixi Biotech Co. Ltd. (Beijing, China). Codeine was purchased from the Material Evidence Identification Center of Ministry of Public Security (Beijing, China). The binding buffer was phosphate buffer saline (PBS, 140 mM NaCl, 2.5 mM KCl, 1.6 mM KH_2_PO_4_, 15 mM Na_2_HPO_4_) with 2.5 mM MgCl_2_. Unless otherwise stated, all experimental samples were annealed prior to use at 95 °C for 5 min followed by quenching on ice for 10 min.

### Isothermal titration calorimetry assay

Isothermal titration calorimetry (ITC) assay was performed on a Nano ITC System (TA Instruments Inc., New Castle, DE, USA). Injections of 10 µL of 250 µM codeine were added from a computer-controlled microsyringe at an interval of 400 s into the DNA aptamer solution (about 8–15 µM, cell volume 1.0 mL) with 300 rpm stirring at 25 °C. Heat produced by codeine dilution was evaluated by performing a control experiment, titrating codeine into the buffer alone. The experimental data were fitted to a theoretical titration curve using software supplied by TA, with ΔG (Gibbs free energy), K_d_ (association constant), and n (number of binding sites per monomer), as adjustable parameters.

### Fluorescence spectra experiments

Fluorescence spectra were collected with a Hitachi F-4600 fluorescence fluorometer (Kyoto, Japan) at 25 °C. For the competition experiments with G-quadruplex probe BPBC, 1 µM DNA sequence and 3 µM BPBC were incubated in the absence or presence of codeine (3 µM) for 30 min at 25 °C. Subsequently, emission spectra were measured from 420 nm to 600 nm with excitation of 408 nm, where excitation and emission slits were set to 5 nm. Single 2-aminopurine substituted codeine binding aptamers were excited at 305 nm and emission spectra were measured from 330 nm to 460 nm, where excitation and emission slits were set to 5 nm.

### Circular dichroism analysis

Circular dichroism analysis were performed on a JASCO spectrometer in 10 mm path length quartz cuvettes. The spectra were recorded with 100 nm/min, at 0.5 nm bandwidth, at 25 °C and normalized by subtraction of the background scan of the buffer.

### Melting assay

For thermal denaturation measurements, folded samples were heated from 25 °C to 85 °C with a heating rate of 1 °C min^−1^ on a JASCO spectrometer in 10 mm path length quartz cuvettes. The UV signal at 265 nm was recorded every 1 °C.

### ^1^H NMR experiments

The DNA used for NMR was purified by RP-HPLC. If not particularly indicated, DNA samples were dissolved in 0.5 mL of PBS buffer containing 10% D_2_O, annealed by heating to 95 °C for 5 min and quenched on ice for 10 min. The final concentration of DNA samples was 200 µM. For the presence of codeine samples, the concentration of codeine was 400 µM. NMR experiments were performed on 600 MHz Varian Bruker spectrometers with data recorded at 25 °C. 1D-proton spectra were acquired with 32 000 data points using 6k accumulated scans due to low sample concentration and processed with an exponential line broadening window function. Solvent suppression was achieved by excitation sculpting. Acquired data were processed and analyzed using Bruker Topspin and MestReNova software.

### Dimethyl sulfate (DMS) footprinting assay

Dimethyl sulfate footprinting experiments were performed according to the protocol developed by Tan *et al*.^58^ 10 picomoles of FAM-labeled CBA-0 or CBA-1 in 200 µL volume were mixed with 4 µL of 10% (v/v) dimethyl sulfate (DMS) in ethanol and incubated for 2 min at room temperature. The reaction was stopped by addition of 200 µL of  stop buffer (0.6 M NaOAc, 0.1 M β-mercaptoethanol, 20 µg sperm DNA). After phenol/chloroform extraction and ethanol precipitation, the DNA was dissolved in 50 µL of  water. Thereafter, 50 µL of  20% (v/v) piperidine in water was added and the samples were heated at 90 °C for 30 min, followed by phenol/chloroform extraction and ethanol precipitation. The precipitated DNA was dissolved in 50% (v/v) deionized formamide in water, denatured at 95 °C for 5 min and resolved on a denaturing 12% polyacrylamide gel.

### Molecular Modeling and docking

Starting structure of (3 + 1) G-quadruplex Form II was generated from the coordinates of the reported NMR structures (PDB entries 2JPZ)^50^. The G·GC triplex structures was also generated from the coordinates of the reported NMR structures (PDB entries 134D)^39^. Molecular modeling was energy-minimized using the Tripos force field for binding energy calculation by the Powell method with a convergence criterion of 0.05 kcal/mol·Å with the maximum iterations set to 500. Molecular docking on CBA-1 was performed using Sybyl-X 2.0 program (Tripos Inc.). All of the hydrogen atoms and charges were added to define the correct configuration and tautomeric states. Docking pocket was generated manually. Then, the structure of the compound codeine built by the Sybyl program was docked into the binding pocket for docking analysis.

### Polymerase Stop Assays

The single stand DNA template CBA-Tem was designed as the following: 5′-ACAGACTAGAAACTTATTGGGGGGGG TGCGGG TAACAGACAACA TGAATGA-3′, which contained part of CBA-1-GGC (underlined sequence). The primer CBA-Pri was FAM-TCA TTC ATG TTG TCT G, with a fluorescein label at its 5′ end. The other part of CBA-1-GGC, CCCCCCCCTGGGTCGGGAGGG AAGGGGGGGGT (named CBA-1-GGC-1) was designed to stop the polymerase reaction. 2 µM CBA-Tem was mixed with 4 µM CBA-Pri and CBA-1-GGC-1 in PBS buffer and heated at 95 °C for 5 min followed by quenching on ice for 10 min. Then the mixture were diluted four times with 1X NEBuffer 2 (50 mM NaCl, 10 mM Tris-HCl, 10 mM MgCl_2_,1 mM DTT) and incubated with 5 µM codeine at room temperature for 30 min. Subsequently, 120 µM dNTP and 5 U DNA polymerase I (Klenow Fragment) was added and followed by polymerization for 5 min at room temperature. Denaturing PAGE was performed to analyze.

## Electronic supplementary material

supplementary information
