# Supplementary material for: Triplex-quadruplex structural scaffold: a new binding structure of aptamer
Source: Sci Rep. 2017 Nov 13;7:15467. doi: 10.1038/s41598-017-15797-5 (PMC5684193; doi:10.1038/s41598-017-15797-5)
Supplement: Supplementary file 1 — supplementary information [file 41598_2017_15797_MOESM1_ESM.pdf]

Supporting Information:

Triplex-quadruplex structural scaffold: a new binding structure of aptamer

Tao Bing [1,2], Wei Zheng [1], Xin Zhang [1,2], Luyao Shen [1,2], Xiangjun Liu [1,2], Fuyi Wang [1,2], Jie Cui [1], Zehui Cao [1] and Dihua Shangguan [1,2,] *

1 Beijing National Laboratory for Molecular Sciences, Key Laboratory of Analytical Chemistry for Living Biosystems, CAS Research/Education Center for Excellence in Molecular Sciences, Institute of Chemistry, Chinese Academy of Sciences, Beijing, 100190, China.

2 University of Chinese Academy of Sciences, Beijing, 100049, China.

* To whom correspondence should be addressed. Tel/Fax:86-10-62528509; Email: sgdh@iccas.ac.cn

Table S1. Codeine binding aptamers and their derivative sequences.

Name	DNA sequence
CBA-0	CCCCCTGGGTCGGGAGGGAAGGGGGTTGGGGGTGCGG
CBA-1	CCCCCTGGGTCGGGAGGGAAGGGGGTTGGGGGTGCGG*G*
CBA-1-strand1	*AAAAA*TGGGTCGGGAGGGAAGGGGGTTGGGGGTGCGGG
CBA-1-strand3	CCCCCTGGGTCGGGAGGGAAGGGGGTT*TATAT*TGCGGG
CBA-1- strand21	*GGGGG*TGGGTCGGGAGGGAA*CCCCC*TTGGGGGTGCGGG
CBA-1-GGC	*CCC*CCCCCTGGGTCGGGAGGGAAGGGGG*GGG*TT*GGG*GGGGGTGCGGG
CBA-1-AAT	*TTT*CCCCCTGGGTCGGGAGGGAAGGGGG*AAA*TT*AAA*GGGGGTGCGGG
CBA-1-GGC-4G4	CCCCCCCCTGGG*G*TCGGG*G*AGGG*G*AAGGGGGGGGTTGGGGGGGGTGCGGG*G*
CBA-1-3G3T	CCCCCTTTTTCTTTATTTAAGGGGGTTGGGGGTGCTTT
CBA-1-G14A	CCCCCTGGGTCGG*A*AGGGAAGGGGGTTGGGGGTGCGGG
CBA-1-G34T	CCCCCTGGGTCGGGAGGGAAGGGGGTTGGGGGT*T*CGGG
CBA-1-G34R	CCCCCTGGGTCGGGAGGGAAGGGGGTTGGGGGTCGGG
CBA-1-G34C35R	CCCCCTGGGTCGGGAGGGAAGGGGGTTGGGGGTGGG
CBA-1-Triplex	CCCCCTAAGGGGGTTGGGGGT
CBA-1-Quadruplex	TGGGTCGGGAGGGAATGCGGG
CBA-1-GGC-1	*CCCCCCCCTGGGTCGGGAGGGAAGGGGGGGGT*
CBA-1-GGC-2	T*GGG*GGGGGTGCGGG
CBA-1-1	*CCCCCTGGGTCGGGAGGGAAGGGGGT*
CBA-1-2	TGGGGGTGCGGG
CBA-1-A19T	CCCCCTGGGTCGGGAGGGTAGGGGGTTGGGGGTGCGG*G*
CBA-1-A19G	CCCCCTGGGTCGGGAGGGGAGGGGGTTGGGGGTGCGG*G*

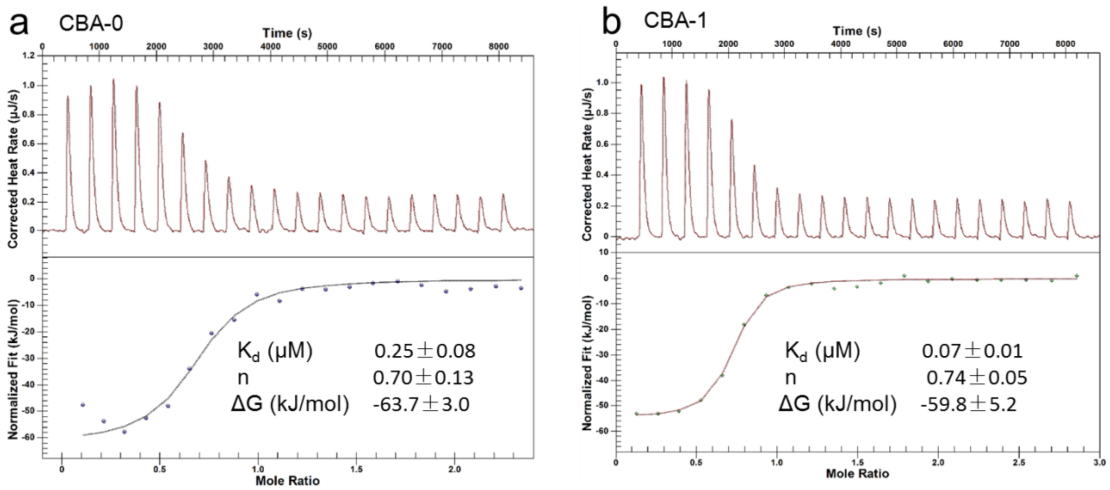

Figure S1. ITC data from the interaction of codeine with CBA-0 (a) or with CBA-1 (b). In each panel, ITC raw profile (at the top) and integrated heat values plotted as a function of the molar ratio (at the bottom) are shown. The errors listed here were from the standard deviation of at least two independent measurement.

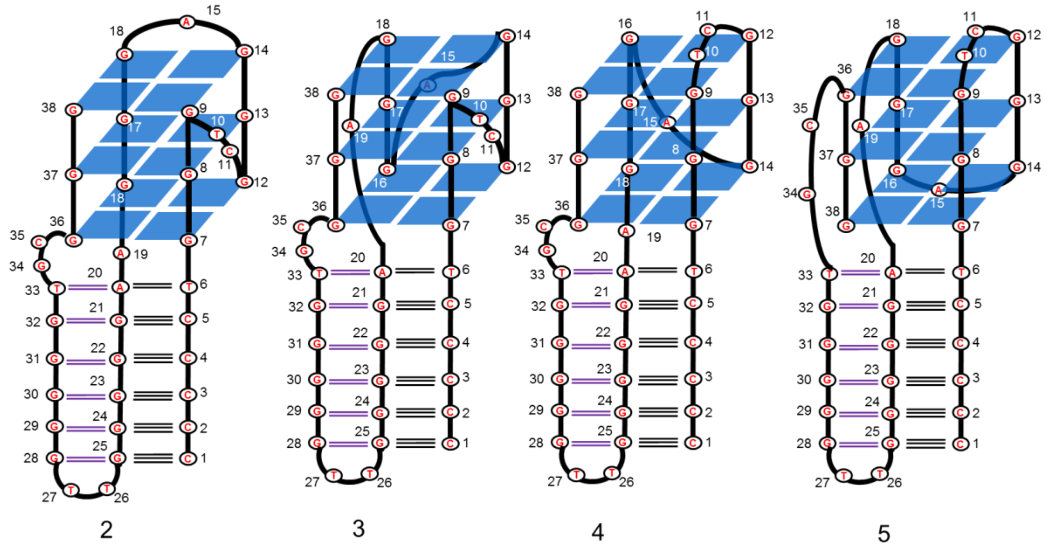

Figure S2. The predicted possible scaffolds 2-6 of CBA-1. The quadruplex moiety: scaffold 2: hybrid; scaffold 3: parallel; scaffold 4: antiparallel; scaffold 5: antiparallel.

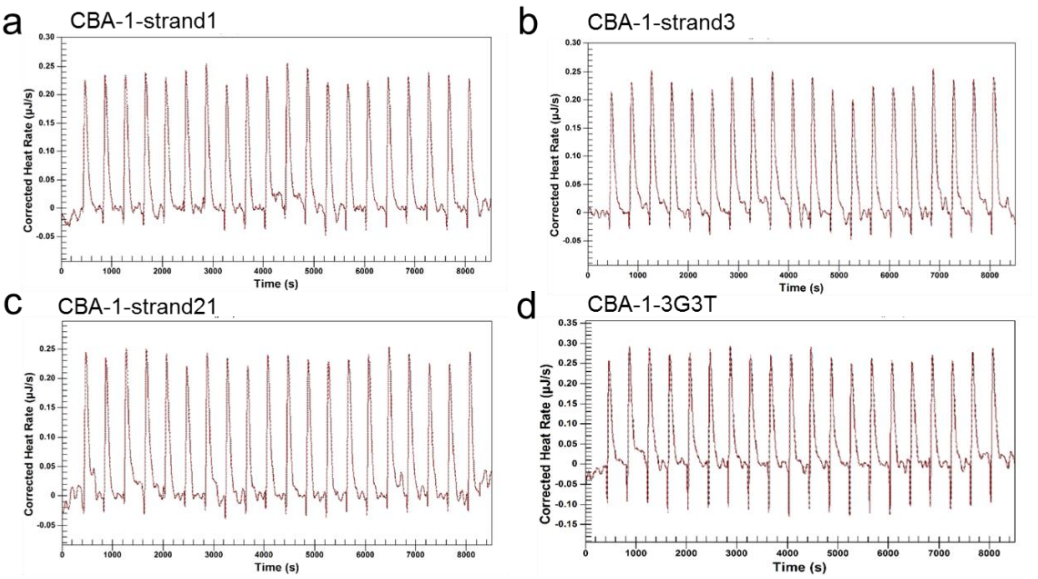

Figure S3. ITC data from the interaction of codeine with CBA-1-strand1 (a), CBA-1-strand3 (b), CBA-1-strand21 (c) and CBA-1-3G3T (d).

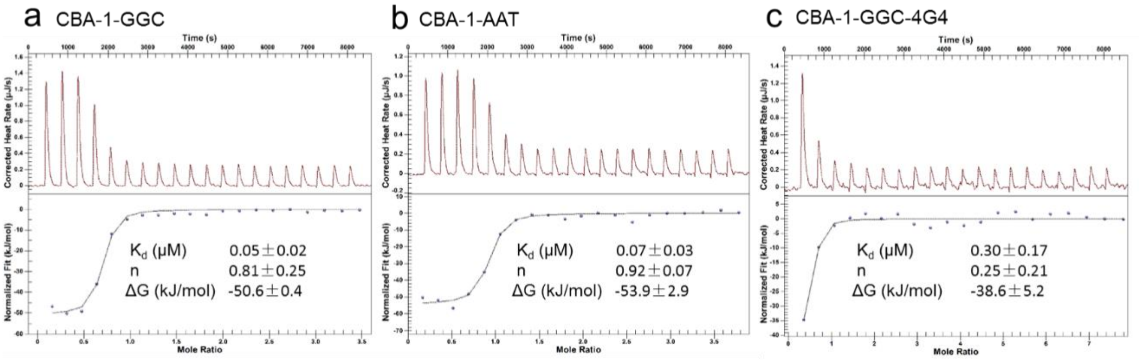

Figure S4. ITC data from the interaction of codeine with CBA-1-GGC (a), CBA-1-AAT (b) and CBA-1-GGC-4G4 (c). The errors listed here were from the standard deviation of at least two independent measurement.

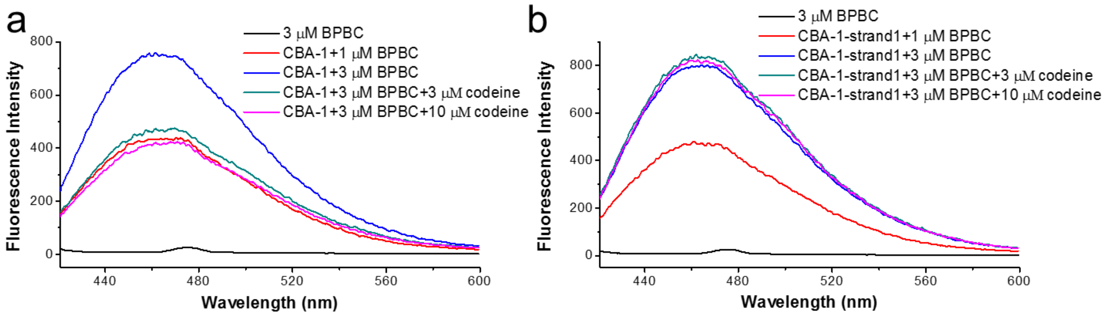

Figure S5. a) Fluorescence spectra of BPBC with 1μM CBA-1 in the absence or presence of codeine. b) Fluorescence spectra of BPBC with 1μM CBA-1-strand1 in the absence or presence of codeine.

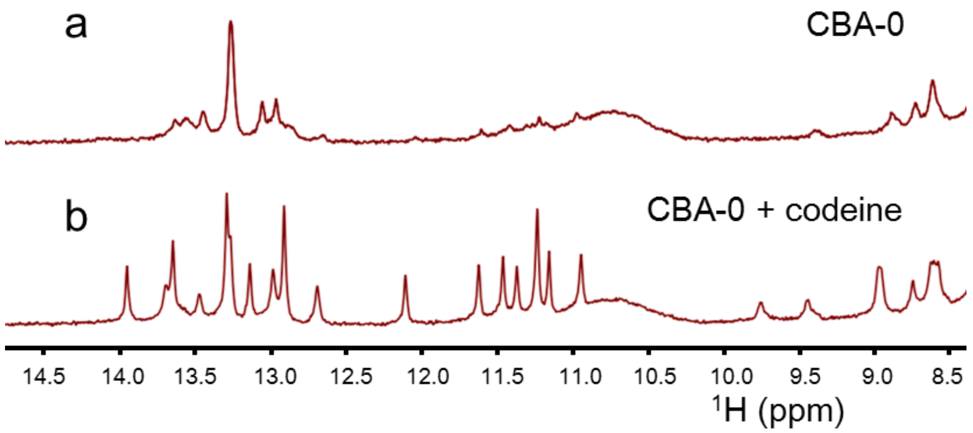

Figure S6. Imino proton regions of 1D 1H NMR spectra of CBA-0(a), CBA-0 in presence of codeine (b).

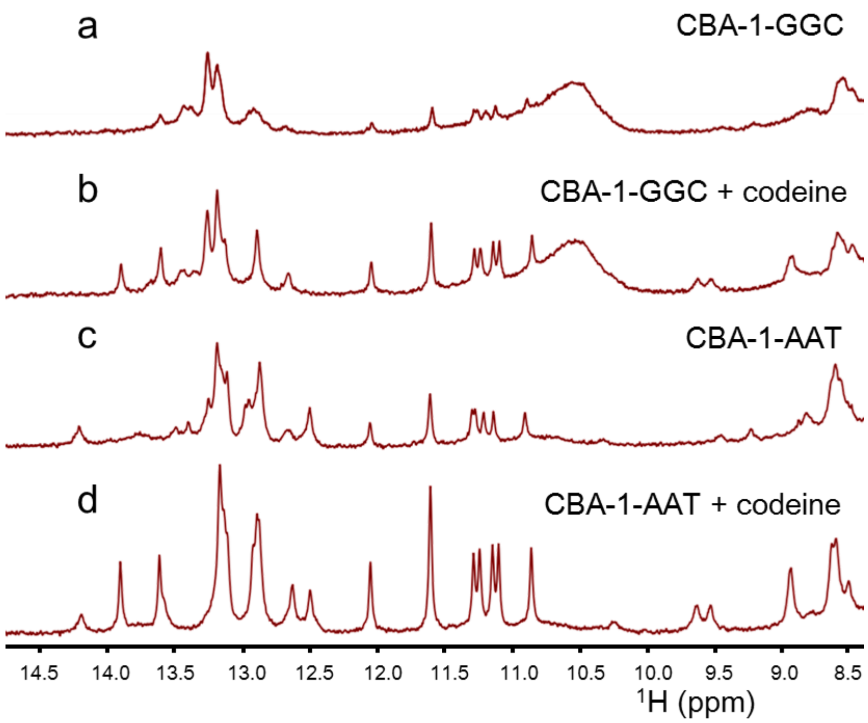

Figure S7. Imino proton regions of 1D 1H NMR spectra of CBA-1-GGC(a), CBA-1-GGC in the presence of codeine (b), CBA-1-AAT(c), CBA-1-AAT in the presence of codeine (d).

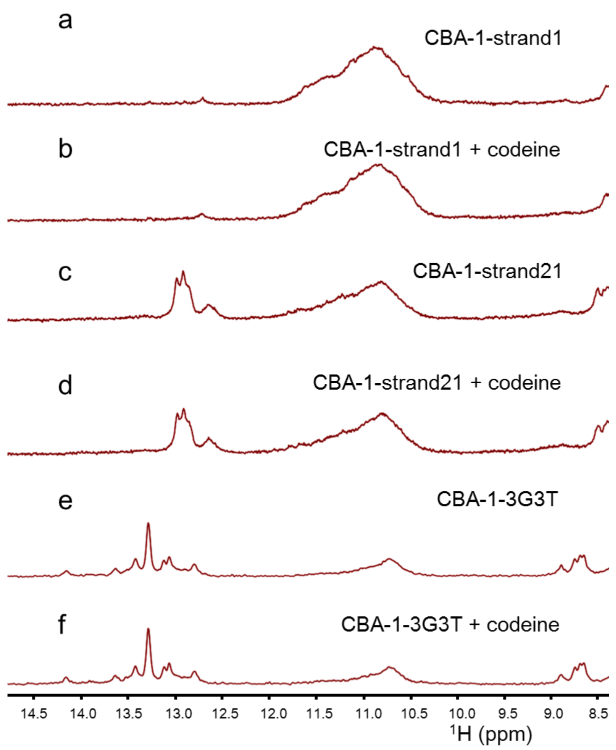

Figure S8. Imino proton regions of 1D 1H NMR spectra of CBA-1-strand1 (a), CBA-1-strand1 in the presence of codeine (b), CBA-1- strand 21(c), CBA-1- strand 21 in the presence of codeine (d), CBA-1-3G3T(e), CBA-1-3G3T in the presence of codeine (f).

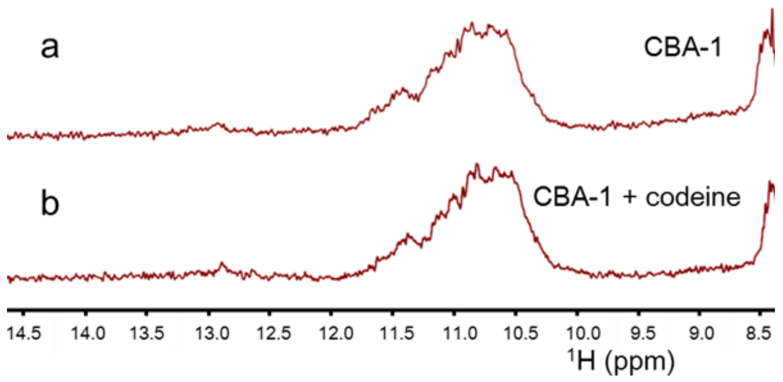

Figure S9. Imino proton regions of 1D 1H NMR spectra of CBA-1(a), CBA-1 in presence of codeine (b) in the buffer containing 15 mM K_2HPO4/ KH_2PO4 and 500 mM KCl, pH7.4.

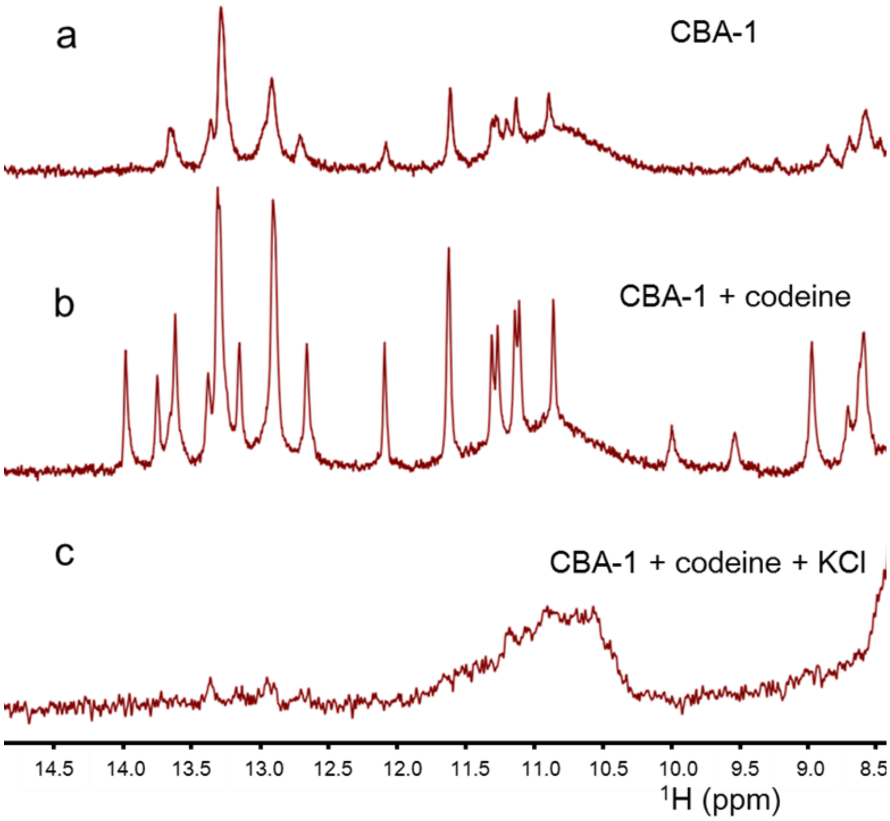

Figure S10. a) Imino proton regions of 1D 1H NMR spectra of CBA-1 in the buffer of 15 mM Na_2HPO4/NaH_2PO4 + 150 mM NaCl, pH 7.4. b) Imino proton regions of 1D 1H NMR spectra of CBA-1 in presence of codeine in the buffer of 15 mM Na_2HPO4/ NaH_2PO4 + 150 mM NaCl, pH 7.4. c) Imino proton regions of 1D 1H NMR spectra of CBA-1 in presence of codeine in the buffer of 15 mM Na_2HPO4/ NaH_2PO4 + 150 mM NaCl + 500 mM KCl, pH 7.4.

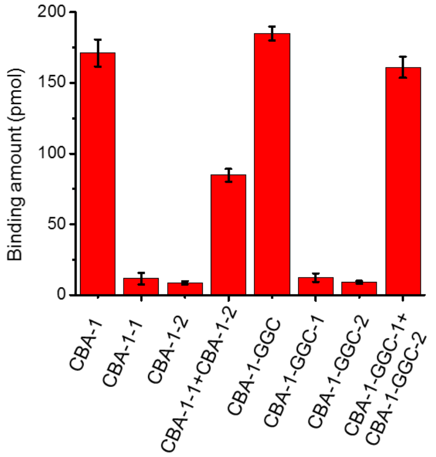

Figure S11. Binding of CBA-1 and CBA-1-GGC subunits on the codeine-coated beads. The DNA was incubated with codeine-coated beads at room temperature, and after eluting the binding DNA using 0.1M NaOH, the binding amount was measured using UV absorbance at 260 nm.

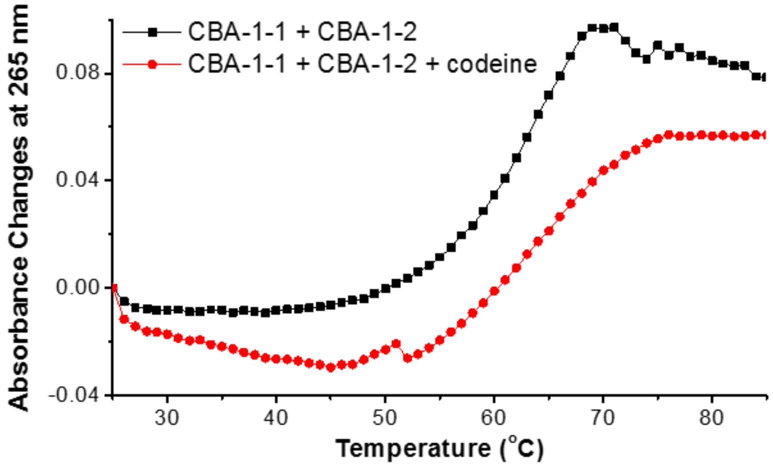

Figure S12. The absorbance thermal denaturation of the split sequences (CBA-1-1 + CBA-1-2) in the absence or presence of codeine at 265 nm.

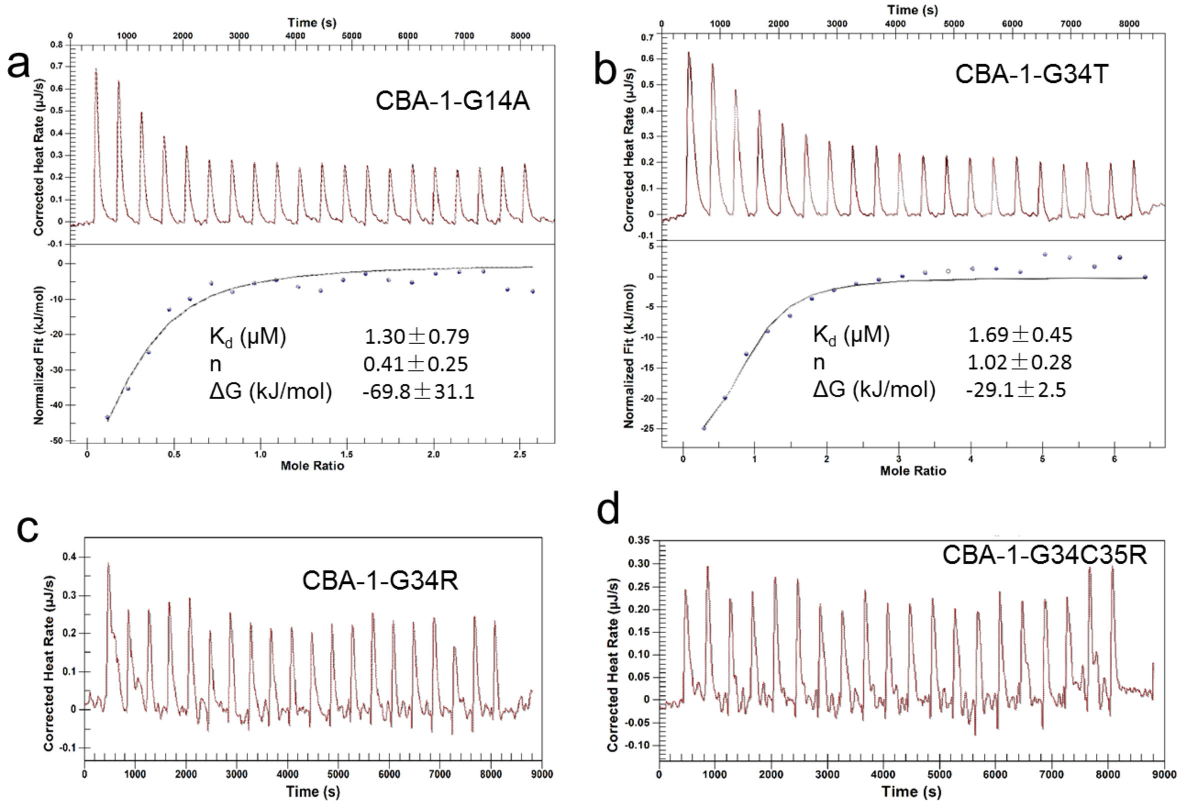

Figure S13. ITC data obtained from the interaction of codeine with CBA-1-G14A (a), CBA-1-G34T (b), CBA-1-G34R (c) and CBA-1-G34C35R (d). The errors listed here were from the standard deviation of at least two independent measurement.

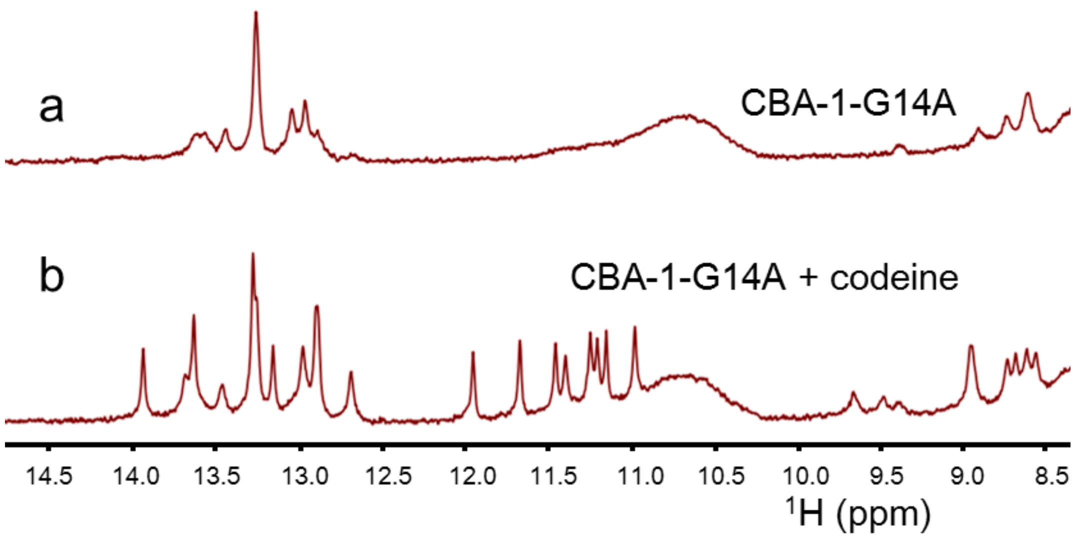

Figure S14. Imino proton regions of 1D 1H NMR spectra of CBA-1-G14A(a), CBA-1-G14A in presence of codeine (b).

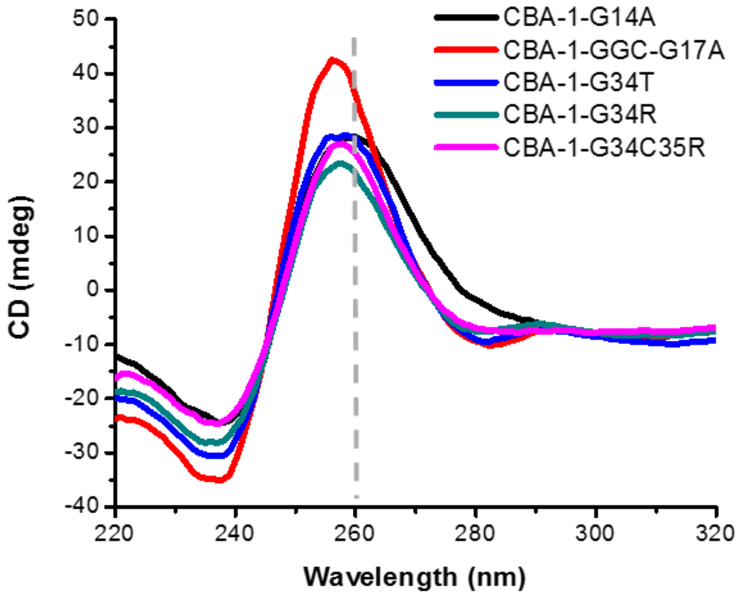

Figure S15. The CD spectra of CBA-1-G14A, CBA-1-GGC-G17A, CBA-1-G34T, CBA-1-G34R and CBA-1-G34C35R.

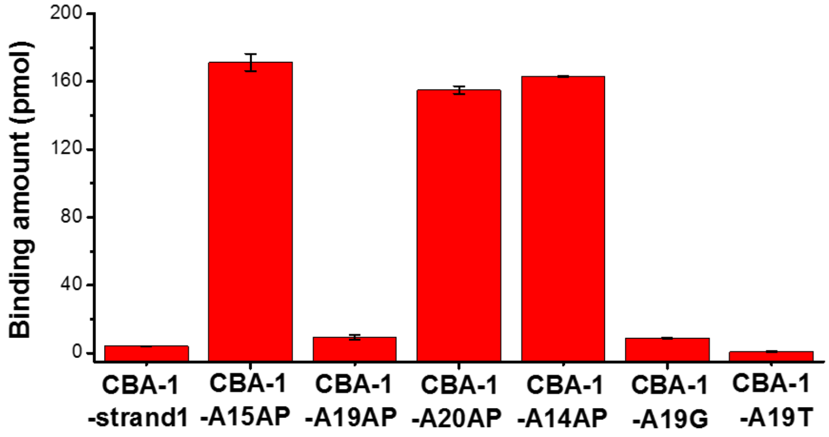

Figure S16. Binding of 2-aminopurine (2AP) substituted codeine binding aptamers, CBA-1-A19G and CBA-1-A19T on the codeine-coated beads. The DNA sequences were incubated with codeine-coated beads at room temperature, and after eluting the binding DNA using 0.1M NaOH, the binding amount was measured using UV absorbance at 260 nm.

a

CBA-1-A15AP CCCCCTGGGTCGGG**X**GGGAAGGGGGTTGGGGGTGCGGG

CBA-1-A19AP CCCCCTGGGTCGGGAGGG**X**AGGGGGTTGGGGGTGCGGG

CBA-1-A20AP CCCCCTGGGTCGGGAGGGA**X**GGGGGTTGGGGGTGCGGG

CBA-1-A14AP CCCCCTGGGTCGG**X**AGGGAAGGGGGTTGGGGGTGCGGG

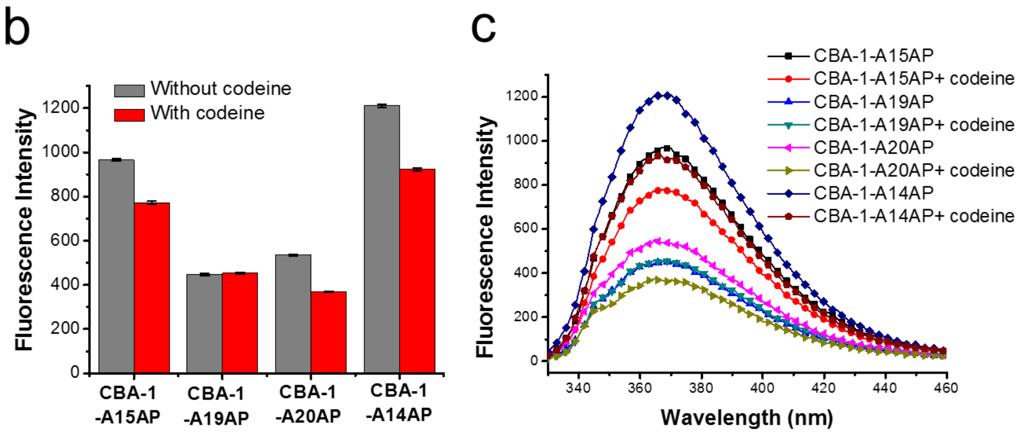

Figure S17. a) Singly 2-aminopurine (2AP) substituted sequences derived from codeine binding aptamer CBA-1. The bold X in each sequence corresponds to the placement of the 2AP in the CBA-1 sequence. b) The fluorescence intensity at 370nm of substituted codeine binding aptamers in the absence or presence 1 μM codeine. c) The fluorescence spectra of 2-aminopurine (2AP) substituted codeine binding aptamers in the absence or presence 1 μM codeine.

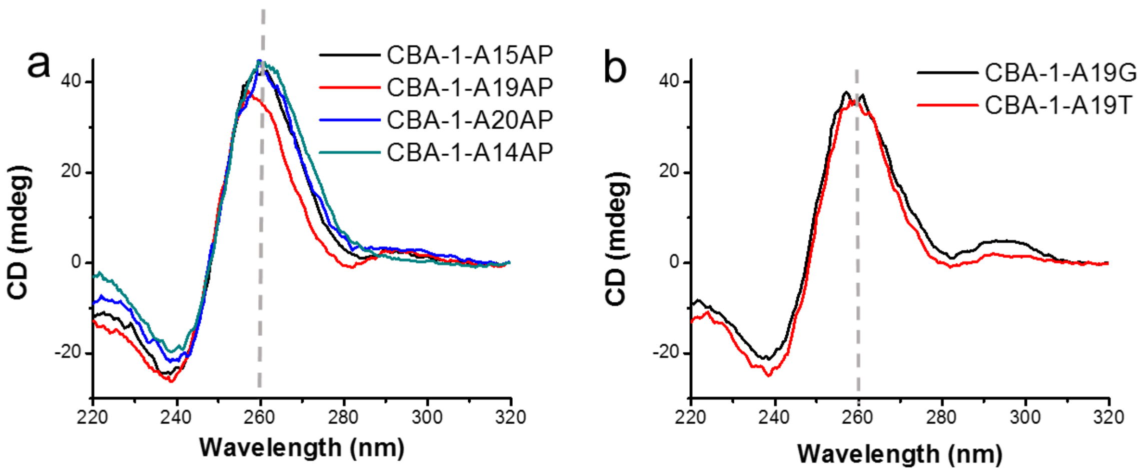

Figure S18. a) CD spectra of the 2-aminopurine (2AP) substituted sequences (CBA-1-A15AP and CBA-1-A19AP, CBA-1-A20AP and CBA-A14AP). b) CD spectra of CBA-1-A19G and CBA-1-A19T.

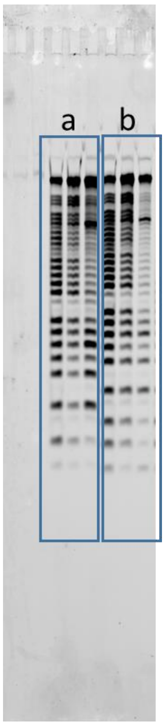

Figure S19. Uncropped full length gel of DMS footprinting assay. The boxed areas are shown in the Figure 4a and b of the main text.

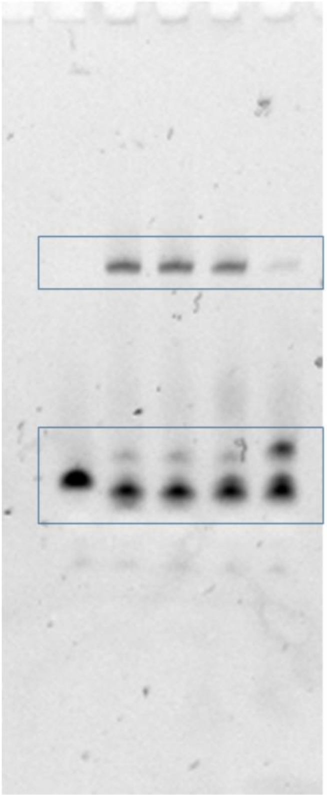

Figure S20. Uncropped full length gel of polymerase stop assays. The boxed areas are shown in Figure 6b of the main text.
